# Indicators of motherhood? Sacral preauricular extensions and notches in identified skeletal collections

**DOI:** 10.1002/oa.3044

**Published:** 2021-10-13

**Authors:** Doris Pany‐Kucera, Michaela Spannagl‐Steiner, Jocelyne Desideri, Katharina Rebay‐Salisbury

**Affiliations:** ^1^ Austrian Archaeological Institute Austrian Academy of Sciences Vienna Austria; ^2^ Department of Anthropology Natural History Museum Vienna Austria; ^3^ Department F.‐A. Forel for Environmental and Aquatic Sciences, Laboratory of Prehistoric Archaeology and Anthropology University of Geneva Geneva Switzerland

**Keywords:** age at first birth, features at the osseous sacrum, heterotopic ossification, identified skeletal collections, parity, pelvis, pregnancy

## Abstract

The sacral preauricular extension (SPE) and sacral preauricular notch (SPN) are morphological changes at the ventral apex of the sacrum. We recently specified their shapes and appearances and suggested a scoring system based on prehistoric Austrian skeletal assemblages. We hypothesized that these specific pelvic changes relate to past pregnancies and parturitions, a hypothesis that we now tested on a subsample of individuals from the Simon Identified Skeletal collection in Geneva (*n* = 62) and the Christ Church, Spitalfields collection in London (*n* = 27) linked to historical information on deliveries. We found SPE and SPN in low frequencies and only in female individuals with at least two children in both collections, and a significant association between the emergence of SPE and first births by 25 years. SPN was found only in two females in the Simon collection, but both with a very high number of recorded parturitions including twin births. Based on these results, we are confident in our assumption that at least SPE, and possibly also SPN, result from increased compression forces at the sacroiliac joint, and especially at the ventrosuperior margin, in recurring (complicated) birth events, the interaction of enhanced pelvic joint mobility that is highest up to age 25, and postural changes related to weight gain during pregnancy. Pelvic shape, dimensions, body proportions, biomechanical issues and hormonal levels may also play a role in their emergence.

## 1 INTRODUCTION

Alterations at pelvic bones have been contextualized with past pregnancies and parturitions and are widely known as “scars of parturition” (Galloway, 1995; Kelley, 1979) or “pelvic scars” (McFadden & Oxenham, 2017). Dorsal pubic pitting and the preauricular sulcus are most often subsumed in these terms. Since not all of them are necessarily scars, e.g. the extended pubic tubercle (Cox, 1989), we prefer the neutral term “pelvic features” (PF) to specify pelvic modifications in this context. Because of the high intra‐ and inter‐population variability, as well as the range of other factors that play a role in the aetiology of pelvic features (e.g., age, stature, pelvic dimensions, biomechanical and musculoskeletal conditions and hormonal influences), the context of origin for most of the “classic” features remains under debate; some studies found relationships to giving birth, while others did not (e.g., Bergfelder & Herrmann, 1980; Cox, 1989; Igarashi et al., 2019; McArthur et al., 2016; McFadden & Oxenham, 2017; Pany‐Kucera et al., 2019, 2021; Perréard Lopreno & Brůžek, 2010; Snodgrass & Galloway, 2003; Stewart, 1970; Suchey et al. 1979; Waltenberger et al., 2020). The focus of this paper is on the occurrence of extensions or notches at the ventral apex (i.e., ventrosuperior margin of the sacral ala) of the sacrum, described within the framework of the ERC‐funded project “The value of mothers to society,” aiming to assess prehistoric women's reproductive and social status. The respective features were first noticed in pelvic remains of skeletons excavated at Austrian sites dating from Neolithic to Iron Age, where 87 male and 126 female individuals were analysed in this regard. In this research, we noticed a previously undescribed feature at the ventral sacral apex in a female from Unterhautzenthal and subsequently encountered similar changes in other prehistoric pelvic remains, but only in females (Pany‐Kucera et al., 2020; Rebay‐Salisbury et al., 2018) and named it “sacral preauricular extension” (SPE). It is an osseous expansion at the level of the terminal line, always located at the ventral apex of the sacral ala. Usually, a subtle line delimits the SPE from the auricular joint surface, which is essential for the differentiation from other changes. The thin, ventrally pointing SPE stands in contrast to marginal osteophytes, which bridge or begin to bridge the joint space at their base in coarse shape from the iliac side. More rarely, a loss of convexity at the same location was noticed, termed “sacral preauricular notch” (SPN). The features are sometimes accompanied by corresponding facets (CF) at the ilium, whereby the SPE causes an imprint and the SPN builds a recess. A systematic approach to analyse the features including the development of a detailed recording scheme was started (Pany‐Kucera et al., 2019).

Sacral changes possibly associated with pregnancy and parturition events in the area of the attachment site of the sacroiliac ligament have been mentioned in previous papers (Andersen, 1986; Cox, 1989; Cox & Scott, 1992; Houghton, 1974; Kelley, 1979; Maass, 2012; Ullrich, 1975). We found the SPE and SPN only in female skeletons, sometimes occurring with other distinctly expressed pelvic features in female pelves (Pany‐Kucera et al., 2021). In collaboration with anatomists from the University of Vienna, a clarification of the causes for the development of the features was pursued. Currently we think that SPE may occur through the mechanism of heterotopic ossification, as it did not seem to be a reparative ossification, whereas SPN probably occurs through epiphyseolysis, both by increased and recurring pressure at this specific location, particularly during complicated labour (Pany‐Kucera et al., 2019). But missing parity information on prehistoric individuals made it impossible to draw reliable conclusions between SPE, SPN and pregnancies or parturitions. This led to the decision to study these specific features in identified skeletal collections with known sex, age at death and information on obstetric histories. We hypothesized that SPE and SPN occur in parous females. The focus of this paper is to test if the incidence and expression of the SPE, the SPN and corresponding facets, may relate to pregnancy and parity in skeletal samples with known obstetric history.

## 2 MATERIAL AND METHODS

Individuals from the Simon Identified Skeletal Collection housed at the University in Geneva (Switzerland) and the Christ Church Spitalfields crypt collection at the Natural History Museum in London (United Kingdom) were analysed. The two selected identified skeletal collections were chosen due to availability of obstetric data for a subset of the females (Abegg & Desideri, 2019; Cox, 1989; Perréard & Eades, 2003; Perréard Lopreno & Brůžek, 2010), which is challenging to find (Maass & Friedling, 2016), even more with females of different age groups.

The Simon Identified Skeletal collection at the University of Geneva comprises 496 individuals of Swiss origin, who had died between the end of the 19th and the early 20th centuries (Abegg & Desideri, 2019; Perréard & Eades, 2003). They stem mostly from a predominantly rural, working‐class, pre‐industrial period population. For all of them, data sheets with basic information like sex and age, and main pathological changes (e.g., fractures) are available, details on the obstetrical history are disposable from 99 females. We selected our skeletal subsample of 62 individuals on‐site (43 females with documented obstetric history, among them 13 nulliparae, 4 primiparae, 26 multiparae; 19 males), mainly based on the preservation status of the pelvis, and excluded those with pathological conditions or fractures affecting the spine, pelvis and lower limbs mentioned in the data sheets (compare differential diagnoses, Pany‐Kucera et al., 2019). The pelves were analysed for the occurrence of SPE, SPN and CF, and only then we obtained information on the number of children, age at first and last birth, birth spacing, and marital status for the female individuals.

The second data acquisition took place at the Natural History Museum in London, and concerned individuals from the Christ Church Spitalfields crypt in East London, who died between 1729 and 1852. They were largely of French origin (Huguenots), settled in Spitalfields due to the silk industry, and were members of the middle class. Obstetric data is available for a subsample of 94 female individuals (total number of individuals in the named sample *n* = 387), and includes number of children, age at first and last birth, birth spacing and marital status (Cox, 1989; Cox & Scott, 1992). We pre‐selected 27 well‐preserved female individuals with documented obstetric data for our analysis (six nulliparae, one primipara and 20 multiparae) based on the descriptions in the extensive publication by Cox (1989). Although we selected the best‐preserved individuals from both groups, some skeletal elements were damaged or missing, which resulted in limited missing data. The on‐site examination was performed blind for the obstetric data. Due to the limited access to the collection, a lack of recording time and the fact that we do have comparable data from 87 male individuals from our prehistoric sites and found no indications of SPE, SPN or CF (Pany‐Kucera et al., 2021), we concentrated on the females in this collection.

**FIGURE 1:**
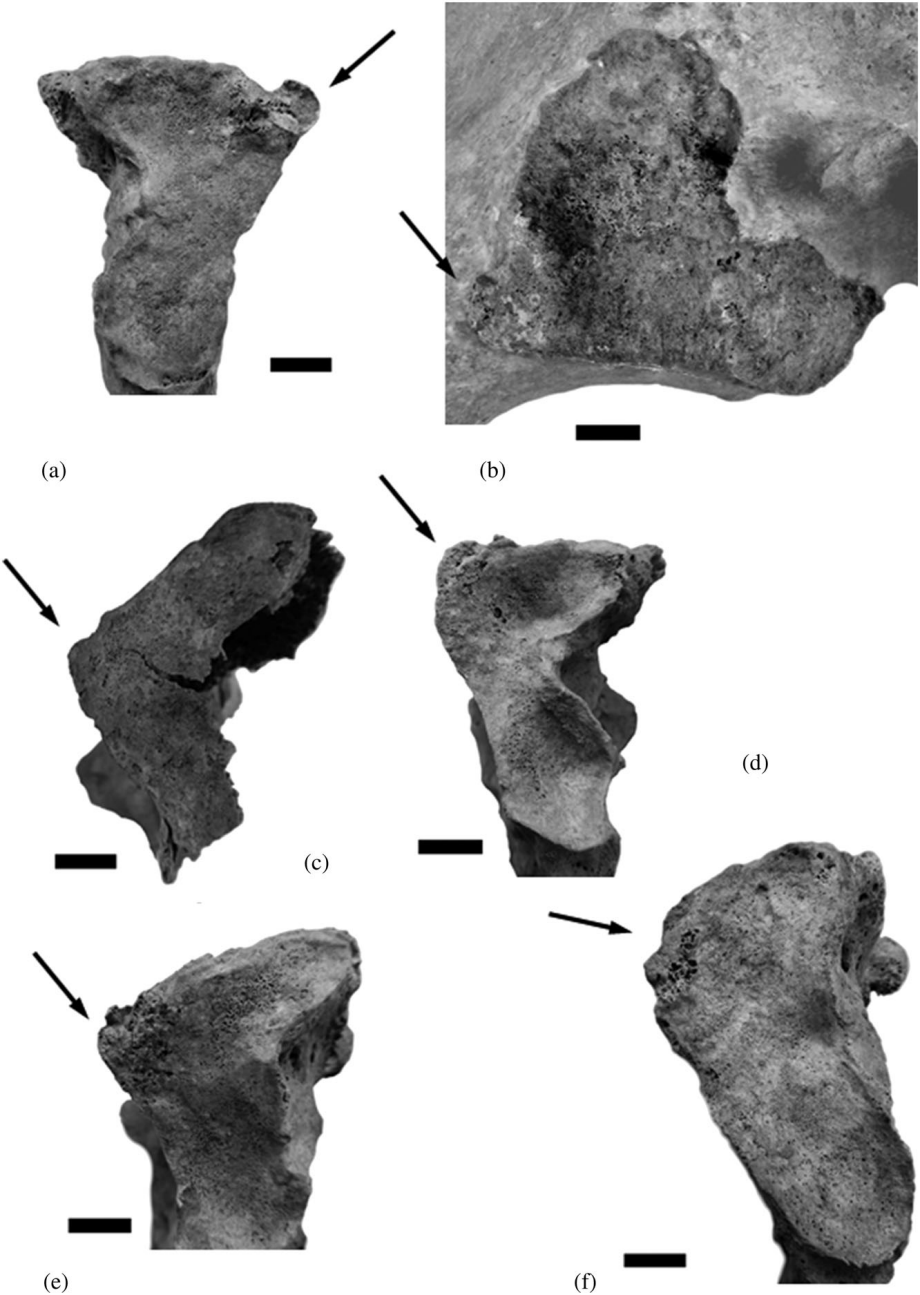
Variants of SPE (a, c–e), CF (b) and SPN (f) in females of the Simon Skeletal Identified Collection, Geneva. Individual number/age at first birth/number of children/age at death: a and b: BIE 32/21 years/5/34 years, c: LSZ 17/24 years/2/50 years, d: PAM 01/18 years/6/82 years, e: PAM 16/22 years/4/71 years and f: STP 02/20 years/9/76 years

**FIGURE 2:**
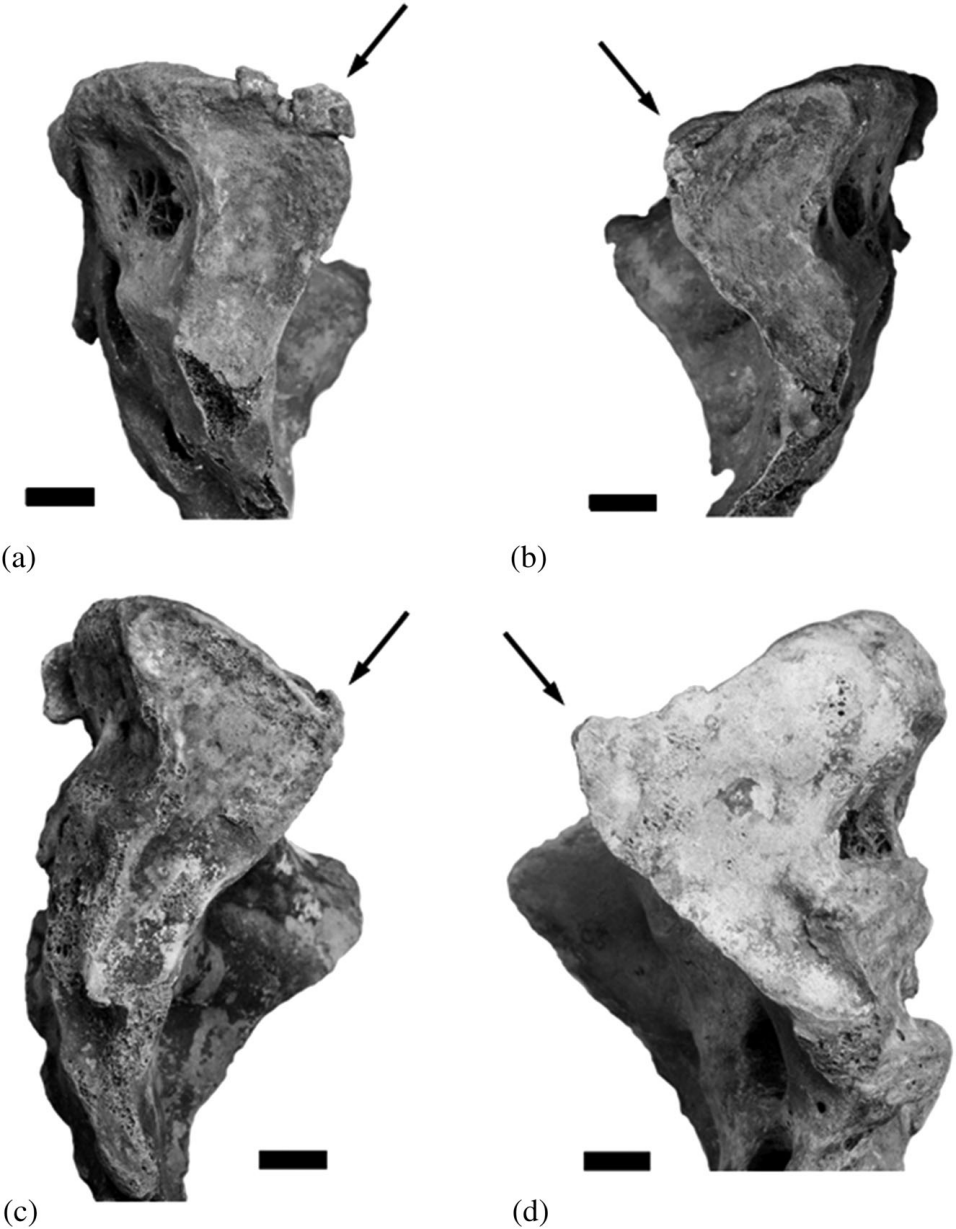
Variants of SPE (a–d) in females of the Christ Church Spitalfields collection, London (courtesy of the trustees of the Natural History Museum, London [February 12th and 13th, 2020]). Individual number/age at first birth/number of children/age at death: a and b (bilateral occurrence): CAS 2327/21 years/3/23 years, c: CAS 2070/19 years/5/35 years and d: CAS 2368/20 years/4/45 years

**FIGURE 3:**
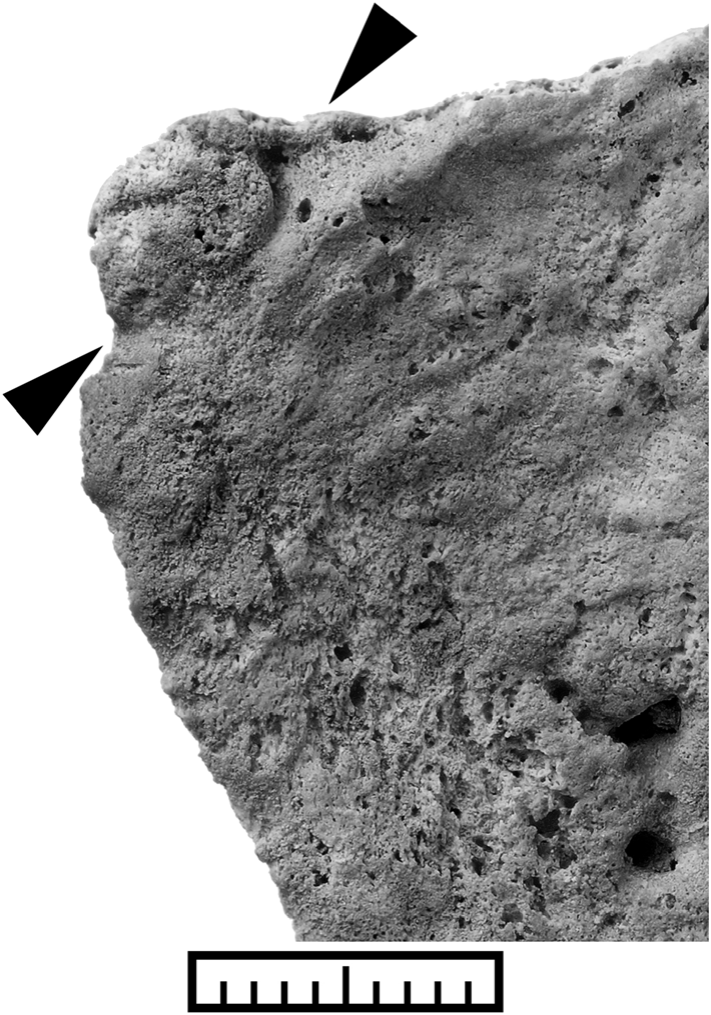
Close‐up of an SPE at the ventrosuperior margin of the left ala ossis sacri. The subtle line detaching it from the auricular surface is well visible here (black arrowheads)

The SPE is defined as a thin, ventrally pointing osseous extension at the ventrosuperior margin of the *ala ossis sacri*, slightly detached by a subtle line (Figures 1, 2, 3) from the sacral auricular surface (1: no bony modification; 2: small to large SPE). SPN is defined as a notch suggesting a loss of convexity at the ventrosuperior margin of the *ala ossis sacri* (1: no bony modification; 2: small to large SPN). Corresponding facets at the iliac side were recorded as 1: no bony modification, and 2: corresponding facet present.

Individuals with signs attributable to DISH or AS were excluded (compare differential diagnosis, Pany‐Kucera et al., 2019). Moreover, the shape of changes at the ventral sacral apex was especially carefully assessed in older individuals, in order to exactly differentiate between possible SPE and marginal osteophytes. The presence of degenerative changes is mentioned in the results when noticed, but details on possible associations with SPE or SPN are discussed elsewhere, as it is beyond the scope here (Pany‐Kucera et al., 2021 forthcoming). Body height was calculated from femur measurements following the recommendations of Ruff et al., 2012. Statistical comparisons were performed in SPSS Version 23. All female individuals from both groups were included to increase the sample size for the calculations (total *n* = 75, of those 19 nulliparae, five primiparae, and 51 multiparae) and compared to the Simon collection males (*n* = 19). We calculated descriptive statistics for both sexes for age and sex, and within the females for parity groups. Results of crosstabs statistical analyses for two independent samples (Monte Carlo confidence level 95%) are shown. Fisher's exact test (in Chi^2^ tests) for small sample sizes are performed and are given in the tables in cases of significant results, to look for possible associations of SPE and SPN with sex, age and body height. Individuals were separated into two age groups (17–39, *n* = 28, and 40+, *n* = 42), and two stature groups (shorter than 155 cm and taller than 155 cm, *n* = 34 respectively). Within the female group (including nulliparae) we tested for the occurrence of SPE and SPN in relation to parity, age at first birth (first birth before or after the age of 25 years), two or more births at 25 years, close birth spacing (≤2 years spacing), and total number of children (three groups: 0–2 children, 3–5 children, 6 or more children). All calculations were first performed for right and left sacral sides separately, and then for the combined right and left side (r_l) variable, consisting of the maximum values from both sides (the latter results are shown in the tables). Counts and expected counts are given in the crosstabs tables, to clarify and strengthen the significant results.

## 3 RESULTS

Descriptive statistic results on age at death and individual numbers in the analysed groups can be found in Table 1. In all the results from the crosstabs analyses, the nulliparous females are included (except for having the first child by 25 years, for obvious reasons), though the results remained significant when excluding them.

**TABLE 1:** Descriptive statistics of individual numbers and age at death in the analysed groups

		Simon collection females	Simon collection males	Spitalfields collection females	Both collections nulliparae	Both collections primi‐ and multiparae	Simon collection nulliparae	Simon collection primi‐ and multiparae	Spitalfields collection nulliparae	Spitalfields collection primi‐ and multiparae
N valid		43	19	27	19	51	13	30	6	21
Age at death (years)	Mean	47.2	36.2	46.9	37.3	50.7	35.4	52.3	41.3	48.5
	Median	44.0	33.0	47.0	35.0	48.0	34.0	50.5	43.5	47.0
	Minimum	18	18	17	17	23	18	23	17	23
	Maximum	82	59	76	62	82	62	82	62	76

### 3.1 Sacral preauricular extension and corresponding facets

In the analysed subsamples, only females (Table 2), and within those, only parous females (Table 3), who had two or more children presented a SPE. The frequency of occurrence of SPE, however, was rather low (c. 6–10%), and no statistically significant difference between the analysed groups regarding the SPE occurrence was found, neither for right or left sides, nor for the combined right and left variable (Fisher's exact test significance (two‐sided) is 0.337 for sex (Table 2) and 0.177 for parity (Table 3)). Corresponding facets at the ilium in the shape of an imprint occurred in both subsamples from the Geneva and London collections with a frequency of c. 4%–6%.

**TABLE 2:** Crosstabs results for the occurrence of SPE with sex for the combined right and left variable (r_l)

Sex * sacral preauricular extension r_l Crosstabulation					
			Sacral preauricular extension r_l		Total
			No SPE present	SPE present	
Sex	Male	Count	17.0	0.0	17.0
		Expected count	15.6	1.4	17.0
	Female	Count	63.0	7.0	70.0
		Expected count	64.4	5.6	70.0
Total		Count	80.0	7.0	87.0
		Expected count	80.0	7.0	87.0

**TABLE 3:** Crosstabs results for the occurrence of SPE with parity for the combined right and left variable (r_l)

Parity * sacral preauricular extension r_l Crosstabulation					
			Sacral preauricular extension r_l		Total
			No SPE present	SPE present	
Parity	Nulliparous	Count	19	0	19
		Expected count	17.1	1.9	19.0
	Parous	Count	44	7	51
		Expected count	45.9	5.1	51.0
Total		Count	63	7	70
		Expected count	63.0	7.0	70.0

In the whole group of parous females from both sites (Simon and Spitalfields, *n* = 51), the occurrence of SPE ranged between 3 in 49 (6.1%), right side, and 5 in 51 (9.8%), left side; including one individual with a bilateral expression, therefore, in the combined variable, *n* = 7. Facets corresponding to SPE at the ilium were found in 2 of 50 females (4%) on the right side, and 3 of 51 (5.9%) on the left side.

A significant association was found in crosstabs calculations between the presence of an SPE and having the first child by 25 years for the combined maximum right and left variable (Table 4, *n* = 47, *p* = 0.034, Fisher's exact test (two‐sided)). No significant results were obtained for right and left sides separately.

**TABLE 4:** Crosstabs and Fisher's exact test results for the occurrence of SPE and age at first birth for the combined right and left variable (r_l)

Age at first birth * sacral preauricular extension r_l Crosstabulation					
			Sacral preauricular extension r_l		Total
			No SPE present	SPE present	
Age at first birth	<25 years	Count	22	7	29
		Expected count	24.7	4.3	29.0
	>25 years	Count	18	0	18
		Expected count	15.3	2.7	18.0
Total		Count	40	7	47
		Expected count	40.0	7.0	47.0

Chi‐Square Tests^a^			
		Exact sig. (2‐sided)	Exact sig. (1‐sided)
Fisher's exact test		0.034	0.025
N of valid cases	47		

^a^ For 2 × 2 crosstabulation, exact results are provided instead of Monte Carlo results.

Moreover, crosstabs results revealed a significant relationship in case of occurrence of SPE and having two or more children by the age of 25 years, when using the combined maximum variable SPE r_l (*n* = 66, *p* = 0.006, Table 5), as well as when calculating right (*n* = 61, *p* = 0.037), and left sides separately (*n* = 65, *p* = 0.049), all results Fisher's exact test (two‐sided).

**TABLE 5:** Crosstabs and Fisher's exact test results for occurrence of SPE and two or more births at 25 years for the combined right and left variable (r_l)

Two or more births at 25 * sacral preauricular extension r_l Crosstabulation					
			Sacral preauricular extension r_l		Total
			No SPE present	SPE present	
Two or more births at 25	Less than two births at 25	Count	42	1	43
		Expected count	38.4	4.6	43.0
	Two or more births at 25	Count	17	6	23
		Expected count	20.6	2.4	23.0
Total		Count	59	7	66
		Expected count	59.0	7.0	66.0

Chi‐Square Tests^a^			
		Exact sig. (2‐sided)	Exact sig. (1‐sided)
Fisher's exact test		0.006	0.006
N of valid cases	66		

^a^ For 2 × 2 crosstabulation, exact results are provided instead of Monte Carlo results.

Clearly insignificant results were found in statistical tests for an association of SPE in females with close birth spacing, or between age groups (Table 6a) and grouped body height (Table 6b).

**TABLE 6a:** Crosstabs results for the occurrence of SPE in age groups for the combined right and left variable (r_l)

Age group * sacral preauricular extension r_l Crosstabulation					
			Sacral preauricular extension r_l		Total
			No SPE present	SPE present	
Age group	17–39	Count	25	3	28
		Expected count	25.2	2.8	28.0
	40+	Count	38	4	42
		Expected count	37.8	4.2	42.0
Total		Count	63	7	70
		Expected count	63.0	7.0	70.0

**TABLE 6b:** Crosstabs results for the occurrence of SPE in body height groups for the combined right and left variable (r_l)

Body height grouped * sacral preauricular extension r_l Crosstabulation					
			Sacral preauricular extension r_l		Total
			No SPE present	SPE present	
Body height grouped	<155 cm	Count	30	4	34
		Expected count	30.5	3.5	34.0
	>155 cm	Count	31	3	34
		Expected count	30.5	3.5	34.0
Total		Count	61	7	68
		Expected count	61.0	7.0	68.0

### 3.2 Sacral preauricular notch and corresponding facets

Like the SPE, the SPN was only found in females of the two samples, and only in multiparous females. A SPN occurred with a frequency of 2 in 51 (3.9%) female individuals who had more than one child by 25 years, at the left *ala sacralis*, and only in the Simon collection.

A statistically significant result was obtained in crosstabs calculations when testing for left side SPN and total number of children (Table 7, *n* = 68, *p* = 0.018, Fisher's exact test [Monte Carlo Sig. two‐sided]). A corresponding recess was observed in one of the two individuals.

**TABLE 7:** Crosstabs and Fisher's exact test results for the occurrence of SPN with the total number of children

Crosstab					
			Left preauricular notch		Total
			No preauricular notch present	Preauricular notch present	
Parity three groups	Zero to two children	Count	31	0	31
		Expected count	30.1	0.9	31.0
	Three to five children	Count	27	0	27
		Expected count	26.2	0.8	27.0
	Six or more children	Count	8	2	10
		Expected count	9.7	0.3	10.0
Total		Count	66	2	68
		Expected count	66.0	2.0	68.0

Chi‐Square tests				
	Value	Monte Carlo sig. (two‐sided)		
		Significance	95% confidence interval	
			Lower bound	Upper bound
Fisher's exact test	6.473	,018^a^	0.015	0.020
N of valid cases	68			

^a^ Based on 10,000 sampled tables with starting seed 1573343031.

### 3.3 Details on the subsample from the identified skeletal Simon collection

Of the 43 females analysed from this collection, 30 have had children; four were primiparae, six females have had two children, and 20 have had more than two children (Table 1, nine females had five or more, and two of them had more than 10, maximum number 15 children). The mean number of children in the selected females was 2.9 (including the nulliparae). Mean age at first birth was 22.7 years (earliest birth at 18 and 19 years), mean age at last birth was 32 years (latest at 43 years); of all females (nulliparae included), 50% have had at least one child, and of the multiparous females, 65.2% have had two or more children by the age of 25 years. The mean body height of the females was c. 158 cm.

The SPE occurred in 1 of 28 (3.6%) multiparous Simon collection females on the right side, and in 3 of 30 on the left side (10%); they have had between two and six children. The youngest among them showed a SPE at the right ala sacralis and died at the age of 34 years; she has had five children between the age of 21 and 30 years (BIE 32, Figure 1a). We observed a corresponding facet at her right ilium (Figure 1b). The three other affected females had a left‐sided SPE (LSZ 17, died aged 50, two children at 24 and 27 years, Figure 1c; PAM 01, died aged 82, six children between the age of 18–41 years, Figure 1d; and PAM 16, died aged 71, four children between the age of 22–34 years, Figure 1e); the first and last‐mentioned individuals showed corresponding facets at the left ilium. Two of the four females with SPE showed age‐related distinct degenerative changes at their lumbar vertebrae (PAM 01 and PAM 16).

Two females in the Simon collection subsample were affected by a SPN at the left sacral ala (STP 02, Figure 1f, and ETA 05); they have had nine (between 20 and 43 years) and 15 children (between 20 and 41 years) and died at the ages of 76 and 78 years, respectively. One of them (ETA 05) showed a small corresponding facet in the shape of a recess at the left ilium. Both had given birth to twins (at 24 and 27 years). They have slight to severe degenerative changes at their lumbar vertebrae.

The mean age for the analysed males from the Simon collection was c. 36 years (Table 1), and their mean body height was c. 169 cm. No relevant pelvic changes such as SPE, SPN or corresponding facets were found in the males (Table 2).

### 3.4 Details on the subsample from the Christchurch Spitalfields collection

The 21 parous females out of the 27 analysed individuals (Table 1) from the Christchurch subsample included one primipara, one woman with two children, and 19 who have had more than two children (seven had five or more, four had 10 or more, maximum number 15 children). The mean number of children in the selected female group was 3.9 (nulliparae included in calculation), mean age at first birth was 26.7 years (earliest at 19 years), mean age at last birth was 36.3 years (latest at 47 years). Of all females (nulliparae included), 33.3% have had at least one child, and 35% of the females have had two or more children by 25 years. The mean body height was c. 151 cm for the selected Spitalfields females.

The SPE occurred in three multiparous females from the Spitalfields collection, the frequency of occurrence is 2 of 21 (9.5%) for each side, with one bilateral expression. The bilateral SPE occurred in a female who had given birth to three children between the age of 21 and 23 (CAS 2327, age at death 23 years (Figure 2a,b)). The individual with the right sided extension has had five children at the age of 19–27 years (CAS 2070, age at death 35 years, Figure 2c), and the individual with the left sided SPE has had four children between the age of 20–38 years (CAS 2368, age at death 45 years, Figure 2d). While the right‐ and the left‐sided SPEs (1 of 21 at each side) showed a corresponding facet at the ilium, the iliac bones of the female with the bilateral SPE did not show these changes. The right femur of CAS 2070 was 3 cm longer than the left one. The oldest of these females showed slight degenerative changes at lumbar vertebrae four and five. A SPN was not found in the Spitalfields subsample.

## 4 DISCUSSION

The results of this study on subsamples from the Simon and the Spitalfields Identified Skeletal collections corroborate our hypothesis that the SPE occurs in relation to multiparity. We found significant associations for SPE within the female group, more precisely for those women who had their first births up to the age of 25 years, and especially those who had minimum two children at this age. No association with close birth spacing or total number of children was detected. We observed no evidence of SPE in nulliparous females or in males. We found no indications that SPE would be more frequent in the age group 40+, or stand in any relationship to body height. Some interesting details were found for the affected women. The three females showing SPE in the Spitalfields subsample had at least two, but up to four children by 25 years of age. Two of them had very close birth spacing. In the Simon collection, three of the four females with SPE had at least two; one had one child by age 25 (and a second at 27).

For SPN in the analysed groups, we obtained significant results with SPN and total number of children, as the two females where it was found had both had a very high number of births. The females with SPN in the Simon collection had two and three children by the age of 25 years, and they were the only ones in this group who have had twins (one at 22/second pregnancy and the other at 26/fourth pregnancy). These mothers of twins were among the few with the overall highest number of children (they have had nine and 15 children respectively). Nevertheless, the occurrence of this feature needs further research.

In the male group from the Simon collection, we did not observe signs of SPE or SPN. The number of analysed males here may appear low, but the results corroborate the absent evidence of SPE or SPN in 87 analysed males from prehistoric contexts (Pany‐Kucera et al., 2019; Pany‐Kucera et al., 2020). In general, shapes and structures of the SPEs and SPNs as well as corresponding facets observed in the Identified Spitalfields and Simon Collections conform well to what we found in our previous studies on prehistoric female skeletons, though their frequencies were higher in the latter (Pany‐Kucera et al., 2019, 2020).

The typical shape of the SPE is thin and points ventrally, directing to the terminal line. A bony expansion causing a disruption of the rounded shape at the ventral apex of the *ala sacralis* is visible (Figures 2a and 2c‐e and 3a‐d); but the SPE never bridges the sacroiliac joint, contrary to changes caused by pathological conditions (Dar & Hershkovitz, 2006; for differential diagnosis compare Pany‐Kucera et al., 2019). The hypothesis that SPE develops by heterotopic ossification seems to be corroborated, as the decisive, most important structure for defining a true SPE is the presence of a subtle line, which delineates it from the sacral auricular surface (Figure 3, Pany‐Kucera et al., 2019). The line is also found in the analysed 19–20th century groups, and is especially noticeable in Figures 2a, 2d and 2e, and 3a–c. In some cases, the line is more difficult to discern (Figures 2c and 3d). Degenerative changes at the sacral auricular facet can make it more difficult to discern SPE from marginal exostoses. However, SPE and SPN exclusively occur at the ventral sacral apex. In rare cases, SPE occurs with a bilateral expression, whereas so far, this was not found for SPN.

We argue that the described features are caused by the conjunction of increased pressure on the ventral sacral apex, weight gain, and increased motion ranges in the sacroiliac joint over the course of multiple gestations and (presumably complicated) parturitions (Pany‐Kucera et al., 2019). After puberty, females have significantly increased levels of oestrogen compared to men. Oestrogen directly influences muscle tissue, tendons, and ligaments, increasing their laxity after puberty (Brunner et al., 1991; Hansen, 2018; Vleeming et al., 2012), particularly up to the age of 25 years, where the highest degree of movement occurs in the sacroiliac joint (Brooke, 1924, Snodgrass & Galloway, 2003, Suchey et al. 1979). This and a different surface architecture allow for a higher range of movement in female sacroiliac joints (Hansen, 2018; Vleeming et al., 2012; Vleeming & Schuenke, 2019). Moreover, increases in hormonal levels, especially of oestrogen, occur in every menstrual cycle with great inter‐individual variability, which also interrelate with nutritional and genetic factors (Hansen, 2018). Increasing relaxin levels in early pregnancy, with a peak in the 12th week, further raise motion ranges in the sacroiliac joints (Borg‐Stein & Dugan, 2007). A change in pelvic posture is induced by weight gain and a shift of the centre of gravity during pregnancy (also initiating gait pattern changes, Ribeiro et al., 2013), which may be maintained after parturition, as levels of oestrogen and relaxin decrease rapidly (Aldabe et al., 2012; Hansen, 2018). All these facts together, in particular the general female hormonal status, especially in pregnant women, and the associated laxity of ligaments and joints together with the exceptionally high pressure on the ventrosacral apex in recurring birth events (or in difficult births), induce the heterotopic ossification mechanism (McCarthy & Sundaram, 2005) and probably contribute to the emergence of the SPE. Although we found no evidence of SPE in the females analysed here and recorded as nulliparae in the documents, however, in general, some of those females may have been pregnant, with stillborn foetuses not necessarily leaving an entry in historical records.

Moreover, we cannot ultimately exclude that SPE would occur in males, but we would rather preclude this possibility based on our hypothesis.

The two females with an SPN both had their first child at 20 years, and more up to 25, which may have affected the relevant metaphysis at the sacrum and therefore cause the SPN (Pany‐Kucera et al., 2019). However, based on our results, we can only conclude on an association with first births and multiple pregnancies up to the age of 25 years for the SPE.

Finally, on that basis and the fact that SPE and SPN generally occur with rather low frequencies, we speculate that some female populations may have more favourable combinations of foetopelvic proportions for giving birth than others, resulting from the interplay of parental genetic makeover, dietary, epigenetic and environmental factors (Fischer & Mitteröecker, 2015; Pavličev et al., 2019; Tague, 2000, Wells et al., 2012). This may significantly influence the occurrence of SPE and SPN in single individuals. More research on larger numbers of individuals from identified skeletal collections with available obstetric data are necessary to corroborate the reliability of the relationship between SPE, SPN and parity.

## 5 CONCLUSION

In this study, we found indications that the occurrence of the SPE and, to a lesser degree, the SPN, emerge with multiple pregnancies and births up to 25 years. We found a statistically significant association between the occurrence of SPE and the age at first births, and giving birth to two or more children up to the age of 25 years. We frequently found the features in young females, and so far, we found no conclusive evidence for a causal relationship of SPE with close birth spacing, age or body height. However, the changes occured in low frequencies here (maximum 10%) and there are multiparae who had their children before or around 20 years that do not show these changes. We therefore conclude that factors such as pelvic shape and dimensions, body proportions, as well as biomechanical and hormonal issues are probably involved in the emergence of the SPE and SPN.

## Data Availability

The data that support the findings of this study are available from the corresponding author upon reasonable request.

## References

[oa3044-bib-0001] Abegg, C. , & Desideri, J. (2019). A probable case of multiple myeloma in a female individual from the Simon Identified Skeletal Collection (late 19th–early 20th century, Vaud, Switzerland). International Journal of Paleopathology, 21, 158–165. 10.1016/j.ijpp.2017.02.001 29776886

[oa3044-bib-0002] Aldabe, D. , Ribeiro, D. C. , Milosavljevic, S. , & Bussey, M. D. (2012). Pregnancy‐related pelvic girdle pain and its relationship with relaxin levels during pregnancy: A systematic review. European Spine Journal, 21(9), 1769–1776. 10.1007/s00586-012-2162-x 22310881 PMC3459115

[oa3044-bib-0003] Andersen, BC . (1986). Parturition scarring as a consequence of flexible pelvic architecture. PhD thesis, Simon Fraser University.

[oa3044-bib-0004] Bergfelder, T. , & Herrmann, B. (1980). Estimating fertility on the basis of birth‐traumatic changes in the pubic bone. Journal of Human Evolution, 9, 611–613. 10.1016/0047-2484(80)90091-3

[oa3044-bib-0005] Borg‐Stein, J. , & Dugan, S. A. (2007). Musculoskeletal disorders of pregnancy, delivery and postpartum. Physical Medicine and Rehabilitation Clinics of North America, 18(3), 459–476. 10.1016/j.pmr.2007.05.005 17678762

[oa3044-bib-0006] Brooke, R. (1924). The Sacro‐iliac joint. Journal of Anatomy, 58(Pt 4), 299–305. 17104023 PMC1249720

[oa3044-bib-0007] Brunner, C. , Kissling, R. , & Jacob, H. A. C. (1991). The effects of morphology and Histopathologic findings on the mobility of the sacroiliac joint. Spine, 16(9), 1111–1117. 10.1097/00007632-199109000-00017 1948401

[oa3044-bib-0009] Cox, M. , & Scott, A. (1992). Evaluation of the obstetric significance of some pelvic characters in an 18th century British sample of known parity status. American Journal of Physical Anthropology, 89(4), 431–440. 10.1002/ajpa.1330890404 1463087

[oa3044-bib-0010] Cox, MJ . (1989). An evaluation of the significance of 'scars of parturition' in the Christ Church Spitalfields sample. Doctoral thesis, University of London.

[oa3044-bib-0043] Dar, G. , & Hershkovitz, I. (2006). Sacroiliac joint bridging: simple and reliable criteria for sexing the skeleton. Journal of Forensic Sciences, 51, 480–483. 10.1111/j.1556-4029.2006.00119.x 16696692

[oa3044-bib-0012] Fischer, B. , & Mitteroecker, P. (2015). Covariation between human pelvis shape, stature, and head size alleviates the obstetric dilemma. Proceedings of the National Academy of Sciences, 112(18), 5655–5660. 10.1073/pnas.1420325112 PMC4426453 25902498

[oa3044-bib-0046] Galloway, A. (1995). Determination of parity from the maternal skeleton: An appraisal. Rivista Antropologia (Roma), 73, 83–98.

[oa3044-bib-0014] Hansen, M. (2018). Female hormones: Do they influence muscle and tendon protein metabolism? Proceedings of the Nutrition Society, 77(1) published online 2017, 32–41. 10.1017/S0029665117001951 28847313

[oa3044-bib-0015] Houghton, P. (1974). The relationship of the pre‐auricular groove of the ilium to pregnancy. American Journal of Physical Anthropology, 41(3), 381–389. 10.1002/ajpa.1330410305 4432925

[oa3044-bib-0017] Igarashi, Y. , Shimizu, K. , Mizutaka, S. , & Kagawa, K. (2019). Pregnancy parturition scars in the preauricular area and the association with the total number of pregnancies and parturitions. American Journal of Physical Anthropology, 171, 1–15. 10.1002/ajpa.23961 PMC7003797 31697408

[oa3044-bib-0045] Kelley, M. A. (1979). Parturition and pelvic changes. American Journal of Physical Anthropology, 51, 541–546. 517643 10.1002/ajpa.1330510405

[oa3044-bib-0044] Maass, P. (2012). The bony pelvis scars of parturition and factors influencing their manifestation. Cape Town: (PhD), University of Cape Town.

[oa3044-bib-0018] Maass, P. , & Friedling, L. J. (2016). Scars of parturition? Influences beyond parity. International Journal of Osteoarchaeology, 26(1), 121–131. 10.1002/oa.2402

[oa3044-bib-0019] McArthur, T. A. , Meyer, I. , Jackson, B. , Pitt, M. J. , & Larrison, M. C. (2016). Parturition pit: The bony imprint of vaginal birth. Skeletal Radiology, 45(9), 1263–1267. 10.1007/s00256-016-2418-3 27270921 PMC5533505

[oa3044-bib-0020] McCarthy, E. F. , & Sundaram, M. (2005). Heterotopic ossification: A review. Skeletal Radiology, 34, 609–619. 10.1007/s00256-005-0958-z 16132978

[oa3044-bib-0021] McFadden, C. , & Oxenham, M. F. (2017). Sex, parity, and scars: A meta‐analytic review. Journal of Forensic Sciences, 63(1), 201–206. 10.1111/1556-4029.13478 28233324

[oa3044-bib-0022] Pany‐Kucera, D. , Spannagl‐Steiner, M. , Argeny, S. , Maurer‐Gesek, B. , Weninger, W. J. , & Rebay‐Salisbury, K. (2019). Sacral preauricular extensions, notches, and corresponding iliac changes: New terms and the proposal of a recording system. International Journal of Osteoarchaeology, 29, 1013–1021. 10.1002/oa.2814

[oa3044-bib-0023] Pany‐Kucera, D. , Spannagl‐Steiner, M. , Waltenberger, L. , Parson, W. , Strobl, C. , Rendl, B. , Janker, L. , Kanz, F. , & Rebay‐Salisbury, K. (2020). Social relations, deprivation and violence at Schleinbach, Lower Austria: Insights from an interdisciplinary analysis of the early Bronze Age human remains. Archaeologica Austriaca, 104, 13–52.

[oa3044-bib-0024] Pany‐Kucera, D. , Spannagl‐Steiner, M. , Maurer‐Gesek, B. , Weninger, W. J. , Rebay‐Salisbury, K. (2021). Sacral preauricular extensions and notches as parts of a “pelvic pattern” may provide information on past pregnancies and parturitions. Forthcoming: Themed issue on pelvic features in *Anthropologischer Anzeiger/HOMO* . 10.1127/anthranz/2021/1455 34761801

[oa3044-bib-0025] Pavličev, M. , Romero, R. , & Mitteroecker, P. (2019). Evolution of the human pelvis and obstructed labor: New explanations of an old obstetrical dilemma. American Journal of Obstetrics and Gynecology., 222, 3–16. 10.1016/j.ajog.2019.06.043 31251927 PMC9069416

[oa3044-bib-0026] Perréard Lopreno, G. , & Brůžek, J. (2010). A well‐evaluated preauricular groove on the hipbone is a very reliable sexual trait but not an indicator of parity. Abstract and Poster at the Annual Meeting of the American Association of Physical Anthropologists.

[oa3044-bib-0027] Perréard Lopreno, G. , & Eades, S. (2003). Une démarche actualiste en paléoanthropologie: La collection de squelettes de référence. In M. Besse , L. I. Stahl Gretsch , & P. Curdy (Eds.), ConstellaSion: Hommage à Alain Gallay (pp. 463–472). Cahs d'archéol. romande; 95.

[oa3044-bib-0029] Rebay‐Salisbury, K. , Pany‐Kucera, D. , Spannagl‐Steiner, M. , Kanz, F. , Galeta, P. , Teschler‐Nicola, M. , & Salisbury, R. B. (2018). Motherhood at early bronze age Unterhautzenthal, Lower Austria. Archaeologia Austriaca, 102, 71–134. 10.1553/archaeologia102s71

[oa3044-bib-0030] Ribeiro, A. P. , João, S. M. A. , & Sacco, I. C. N. (2013). Static and dynamic biomechanical adaptations of the lower limbs and gait pattern changes during pregnancy. Women's Health, 9(1), 99–108. 10.2217/WHE.12.59 23241158

[oa3044-bib-0031] Ruff, C. B. , Holt, B. M. , Niskanen, M. , Sladék, V. , Berner, M. , Garofalo, E. , Garvin, H. M. , Hora, M. , Maijanen, H. , Niinimäki, S. , Salo, K. , Schuplerová, E. , & Tompkins, D. (2012). Stature and body mass estimation from skeletal remains in the European Holocene. American Journal of Physical Anthropology, 148(4), 601–617. 10.1002/ajpa.22087 22639191

[oa3044-bib-0032] Snodgrass, J. , & Galloway, A. (2003). Utility of dorsal pits and pubic tubercle height in parity assessment. Journal of Forensic Science, 48, 1226–1230. 10.1520/JFS2003027 14640264

[oa3044-bib-0034] Stewart, T. D. (1970). Identification of the scars of parturition in the skeletal remains of females. In T. D. Stewart (Ed.), Personal identification in mass disasters (pp. 127–135). National Museum of Natural History. 10.5479/sil.30678.39088001440254

[oa3044-bib-0035] Suchey, J. M. , Wiseley, D. V. , Green, R. F. , & Noguchi, T. T. (1979). Analysis of dorsal pitting in the Os pubis in an extensive sample of modern American females. American Journal of Physical Anthropology, 51(4), 517–539. 10.1002/ajpa.1330510404 517642

[oa3044-bib-0036] Tague, R. G. (2000). Do Big Females Have Big Pelves? American Journal of Physical Anthropology, 112, 377–393. 10.1002/1096-8644(200007)112:3<377::AID-AJPA8>3.0.CO;2-O 10861354

[oa3044-bib-0037] Ullrich, H. (1975). Estimation of fertility by means of pregnancy and childbirth alterations at the pubis, the ilium and the sacrum. Ossa, 2(1), 23–39.

[oa3044-bib-0038] Vleeming, A. , Schuenke, M. D. , Masi, A. T. , Carreiro, J. E. , Danneels, L. , & Willard, F. H. (2012). The sacroiliac joint: An overview of its anatomy, function and potential clinical implications. Journal of Anatomy, 221(6), 537–567. 10.1111/j.1469-7580.2012.01564.x 22994881 PMC3512279

[oa3044-bib-0039] Vleeming, A. , & Schuenke, M. (2019). Form and force closure of the sacroiliac joints. Physical Medicine and Rehabilitation [Epub Ahead of Print]., 11. 10.1002/pmrj.12205 31218826

[oa3044-bib-0040] Waltenberger, L. , Pany‐Kucera, D. , Rebay‐Salisbury, K. , & Mitteroecker, P. (2020). The association of parturition scars and pelvic shape: A geometric morphometric study. American Journal of Physical Anthropology, 174, 519–531. 10.1002/ajpa.24196 33295660 PMC7898533

[oa3044-bib-0041] Wells, J. C. K. , DeSilva, J. M. , & Stock, J. T. (2012). The obstetric dilemma: An ancient game of Russian roulette, or a variable dilemma sensitive to ecology? American Journal of Physical Anthropology, 149(S55), 40–71. 10.1002/ajpa.22160 23138755

